# A Potential Therapeutic Role of Myoinositol in the Metabolic and Cardiovascular Profile of PCOS Iranian Women Aged between 30 and 40 Years

**DOI:** 10.1155/2016/7493147

**Published:** 2016-08-25

**Authors:** Saghar Salehpour, Leila Nazari, Sedighe Hoseini, Nasrin Saharkhiz, Fatemeh Ghazi, Mohammad Reza Sohrabi

**Affiliations:** Department of Obstetrics and Gynecology, Preventive Gynecology Research Center, Shahid Beheshti University of Medical Sciences, Tehran, Iran

## Abstract

*Introduction*. Polycystic ovary syndrome (PCOS) is a common disorder in reproductive age. This pilot study investigated the effects of myoinositol (MI) treatment on metabolic and cardiovascular profile in PCOS women over 30 years of age.* Methods*. Between 2015 and 2016, 50 women with diagnosis of PCOS by the Rotterdam Criteria were included in the study. All women received MI 2 g plus 200 *μ*g of folic acid (Inofolic, Health Parsian, Iran; twice daily) for 3 months. Baseline and 3-month serum samples were taken after an overnight fast to evaluate the insulin resistance index (HOMA-IR), fasting glucose, and the levels of triglyceride, total cholesterol, high-density lipoprotein (HDL), low-density lipoprotein (LDL), homocysteine, systolic blood pressure, and diastolic blood pressure. Participants' weight was measured before and after treatment and body mass index (BMI) was calculated.* Results*. The data showed a significant improvement in the serum level of insulin sensitivity and a reduction of cholesterol, LDL, and homocysteine after three months of treatment. Furthermore, blood pressure was significantly reduced in the treated patients. Three participants became pregnant during treatment.* Conclusion*. Results showed that supplementation with MI and folic acid in PCOS patients over 30 years of age could decrease the risk of cardiovascular problems by normalizing the metabolic profile.

## 1. Introduction

Polycystic ovary syndrome (PCOS) is a common disorder in reproductive age which affects up to 20% of women. Polycystic ovaries on ultrasound, menstrual irregularity, and hyperandrogenism which can lead to acne, alopecia, hirsutism, insulin resistance, android obesity, dyslipidemias, infertility, and early pregnancy loss characterize it [1–3]. The main alteration induced by PCOS seems to be an increased androgen synthesis and secretion at ovarian level; insulin resistance and obesity may exacerbate this alteration, explaining the link between PCOS and obesity and insulin resistance [4]. Insulin resistance is a key factor not only in obese women but also in more than half of normal-weight women, indicating that, in the most severe cases, metabolic syndrome (MS) may be already established: this pathway can be responsible for the changing picture of PCOS throughout the life. These new findings imply that PCOS itself cannot be considered anymore as a typical disorder of young and fertile age, but it can be considered as an important condition in the different phases of life, from adolescence to postmenopause [5].

Indeed, more than half of women with PCOS suffer from insulin resistance, a condition that could cause the development of other related diseases such as MS, obesity, gestational diabetes, type 2 diabetes, and cardiovascular disease (CVD). Due to all the possible implications that PCOS can have in the medium and long term, great attention has been focused on the management of PCOS women as patients at risk of severe cardiovascular and metabolic diseases. The prevalence of at least one feature of MS has been found to be more than 50% in adult PCOS women, commonly constituted by lipid alterations, such as HDL decrease and LDL and total cholesterol increase. Furthermore, typical signs of increased CVD risk are present (increase of systolic pressure, increase of diastolic pressure, etc.) [4, 5].

Hence, the clinical management of PCOS women and prevention of MS and CVD are important aspects in medicine [6–8], especially in those patients over 30 years of age.

Hormonal contraceptives, insulin-sensitizing drugs such as metformin, thiazolidinediones, statins, orlistat, and N-acetylcysteine treatments were performed for women with PCOS but their usage is limited due to some contraindications and side effects [9–13].

Recent studies have proven that alteration in the insulin pathway could be due to defected inositolphosphoglycans (IPGs) second messengers. IPGs are directly involved in activating the glucose metabolism; therefore, PCOS women seem to show a defect in tissue availability or altered metabolism of inositol that could bring about insulin resistance [14]. MI is the most abundant isoform of inositol in nature and in the human body and it belongs to the vitamin B complex group with insulin-like action. It is hypothesized that there is a correlation between reduction of MI and insulin resistance. Several studies suggested the efficacy of MI in reducing the insulin resistance and improving the ovarian function in PCOS women, but its efficacy to ameliorate the cardiovascular and metabolic profile of adult PCOS women has not been fully elucidated [14–16].

The aim of this pilot study was to investigate the effects of MI treatment based on the cardiovascular and metabolic profile of PCOS Iranian women.

## 2. Methods

Fifty women in reproductive age, between 30 and 40 years, with diagnosis of PCOS by Rotterdam's criteria (European Society of Human Reproduction and Embryology/American Society for Reproductive Medicine; ESHRE/ASRM 2003) were enrolled into the study in the gynecologic clinic of Taleghani Hospital between 2015 and 2016. All participants signed a written informed consent. Ethical committee of Shahid Beheshti University of Medical Sciences (SBMU) approved the study.

Exclusion criteria were absence of enzymatic adrenal deficiency and/or other endocrine diseases and no hormonal treatments in the previous 6 months.

All the women received MI 2 g plus 200 *μ*g of folic acid twice daily (Inofolic, Health Parsian, Iran) for 3 months. Patients were not instructed to follow any diet or lifestyle modification. Baseline and 3-month serum samples were taken after an overnight fast to determine level of fasting glucose, insulin resistance (HOMA), homocysteine, triglyceride, total cholesterol, high-density lipoprotein (HDL), and low-density lipoprotein (LDL). Systolic pressure and diastolic pressure were also checked at baseline and after treatment in all the patients. Participants' weight was measured before and after treatment and BMI was calculated.

Insulin sensitivity was calculated as glucose-to-insulin ratio. HOMA index was calculated as [basal glucose] × [basal insulin]/22.5.

The homocysteine levels were detected using a fluorometric HPLC protocol.

Sample size was determined after consideration of type 1 statistical error <5% and type 2 statistical error <20%.

Results were shown as mean ± SD. Statistical analysis was performed using the SPSS 21.0 statistical software package (SPSS Inc., Chicago, IL, USA). Plasma triglycerides were analyzed with Mann–Whitney *U* test and other data were analyzed with *t*-test for quantitative variables and Chi square test for qualitative variables. A *p* value of 0.05 was considered significant.

## 3. Results

Fifty participants were enrolled in this pilot study. Forty-six participants were able to complete the study and their data were included in the final analysis. Four subjects refused the study as follows: one subject discontinued intervention because of dry mouth and three subjects became pregnant during the study.

Data are shown as mean ± SD.  Table 1 provides a summary of the baseline characteristics and outcomes data after a treatment of 3 months.

Significant results were observed in all the patients for what concerned the hormonal and metabolic parameters under evaluation.

As shown in Table 1, there was a significant decrease in the fasting glucose, HOMA index, cholesterol, and LDL. For what concerned cardiovascular parameters, a significant decrease was observed in the homocysteine levels and in the levels of systolic pressure and diastolic pressure (Table 1).

Furthermore, no significant difference was found in the HDL and triglycerides levels. Even the BMI was not influenced by the treatment.

Among 38 participants with irregular menstrual cycles including oligomenorrhea, 60.5% had regular menstrual cycle after treatment. Three participants were pregnant spontaneously during the study just with MI intake and without any other intervention.

No relevant side effects were reported during and after the treatment among the patients.

## 4. Discussion

The present study shows that supplementation with MI and folic acid positively affects metabolic parameters (i.e., insulin sensitivity) and the cardiovascular profile of women over 30 years of age affected by PCOS.

PCOS is a common endocrine disorder in women in reproductive age. Ovarian dysfunction, androgen excess, insulin resistance, and obesity may increase the risk of infertility, type 2 diabetes, CVD, psychological disorders, and cancer. Most of the PCOS women present the typical signs of increased cardiovascular risk and metabolic disorders. In particular, obesity itself has a significant impact on the severity of these manifestations. Therefore, adequate management in PCOS is necessary to decrease the morbidities [17–19].

Not only is PCOS relevant during the young and fertile age, but also elder women can present some related signs and symptoms, which could predict an increased risk of MS, with sequent manifestations during the menopausal period [5].

MI, previously classified as belonging to the vitamin B class, is commonly used in PCOS treatment without any reported and relevant side effects [20]. The efficacy of MI in reduction of insulin resistance, hirsutism, and hyperandrogenism and improvement of ovarian function was reported in several studies but only a few of them have directly checked the outcome in nonyoung PCOS women, in particular for what concerned the efficacy on the lipid profile and cardiovascular profile [14, 15, 21, 22].

Papaleo and coworkers designed a trial to determine the effects of MI on oocyte quality in PCOS women undergoing intracytoplasmic sperm injection (ICSI) cycles. The data showed the reduction of degenerated oocyte and germinal vesicles at ovum pick-up after MI treatment [23].

Gerli and coworkers reported a significant increase in HDL level after MI treatment in PCOS women but the reduction of LDL level was not significant [24].

Furthermore, very recent evidence has been published regarding the positive role of inositol in the metabolic profile of PCOS. An international consensus conference confirmed that MI is effective in the restoration of insulin signaling and some other important parameters negatively influenced by this syndrome [25].

Moreover, besides MI, a great interest in some natural agents has been increased in the recent years. In particular, cocoa polyphenols have shown a strong beneficial action on the cardiovascular profile of different models [26, 27]. Interestingly, there is some evidence showing that the combination of MI and cocoa polyphenols in postmenopausal women with MS was able to restore their metabolic and cardiovascular profile [28, 29]. Therefore, it could be speculated that PCOS women could take advantage of MI supplementation from the adolescence up to the menopausal age. This is the first trial of MI in PCOS women in our country. The prevalence of PCOS in Iranian population is around 15% [30]. We designed the trial to evaluate the efficacy of MI on the metabolic and cardiovascular profile of Iranian PCOS women over 30 years of age. Our data suggested that MI is effective in improving the insulin sensitivity, the lipid parameters, and the blood pressure after a short treatment of three months. However, MI treatment did not influence the BMI of the patients; therefore, we could speculate that restoration of the metabolic profile is mainly due to the insulin-sensitizing action of MI and not due to a different diet or lifestyle during the treatment period. Hence, long-term administration of MI may be helpful to decrease the risk of serious CVD with a possible improvement of fertility without parallel administration of hormonal treatments.

## 5. Conclusion

It seems that use of MI plus folic acid in PCOS patients aged between 30 and 40 years could decrease the risk of cardiovascular and metabolic problems by normalizing the insulin, the lipid profile, and the blood pressure profile. Anyway, further studies with longer treatments and bigger population should be conducted to prove the use of this new agent in these target patients. If further evidence will be assessed, myoinositol combined with folic acid might be considered to be one of the choices for the treatment of PCOS women at risk of metabolic syndrome and consequently for the reduction of their cardiovascular risk.

## Figures and Tables

**Table 1:** Patients' characteristics and outcomes data.

	Before treatment	After treatment	*p* value
Fasting glucose (mg/dL ± SD)	82.06 ± 4.7	79.46 ± 4.2	0.011^*∗*^
HOMA IR	3.4 ± 2.5	2.6 ± 2.5	0.001^*∗*^
Homocysteine (*µ*mol/L)	9.10 ± 2.6	8.54 ± 2.39	0.008^*∗*^
Cholesterol (mg ± SD)	174.7 ± 41.0	160.3 ± 29.5	0.005^*∗*^
LDL (mg ± SD)	94 ± 26.5	86.3 ± 18.3	0.017^*∗*^
HDL (mg ± SD)	47.7 ± 6.5	48.6 ± 6.9	NS
Triglyceride (mg ± SD)	108.2 ± 55.3	110.7 ± 55.3	NS
Systolic blood pressure (mmHg)	130 ± 1.9	126 ± 1.9	0.002^*∗*^
Diastolic blood pressure (mmHg)	89 ± 1.1	83 ± 3.1	0.001^*∗*^
BMI (kg/m^2^± SD)	26.9 ± 3.4	26.8 ± 3.4	NS

^*∗*^Statistically significant differences was observed.

HOMA IR: insulin resistance; LDL: low-density lipoprotein; HDL: high-density lipoprotein; BMI: body mass index; SD: standard deviation.

## References

[1] Vizza L., Smith C. A., Swaraj S., Agho K., Cheema B. S. (2016). The feasibility of progressive resistance training in women with polycystic ovary syndrome: a pilot randomized controlled trial. *BMC Sports Science, Medicine and Rehabilitation*.

[2] Suvarna Y., Maity N., Kalra P., Shivamurthy M. (2016). Comparison of efficacy of metformin and oral contraceptive combination of ethinyl estradiol and drospirenone in polycystic ovary syndrome. *Journal of the Turkish German Gynecological Association*.

[3] Motta A. B. (2012). The role of obesity in the development of polycystic ovary syndrome. *Current Pharmaceutical Design*.

[4] Minozzi M., Costantino D., Guaraldi C., Unfer V. (2011). The effect of a combination therapy with myo-inositol and a combined oral contraceptive pill versus a combined oral contraceptive pill alone on metabolic, endocrine, and clinical parameters in polycystic ovary syndrome. *Gynecological Endocrinology*.

[5] Pasquali R., Gambineri A. (2006). Polycystic ovary syndrome: a multifaceted disease from adolescence to adult age. *Annals of the New York Academy of Sciences*.

[6] Ou H. T., Chen P. C., Wu M. (2016). Effect of metformin by employing 2-hour postload insulin for measuring insulin resistance in Taiwanese women with polycystic ovary syndrome. *Journal of the Formosan Medical Association*.

[7] Kumar A. N., Naidu J. N., Satyanarayana U., Ramalingam K., Anitha M. (2016). Metabolic and endocrine characteristics of Indian women with polycystic ovary syndrome. *International Journal of Fertility & Sterility*.

[8] Cussons A. J., Watts G. F., Burke V., Shaw J. E., Stuckey B. G. A. (2008). Cardiometabolic risk in polycystic ovary syndrome: a comparison of different approaches to defining the metabolic syndrome. *Human Reproduction*.

[9] Salehpour S., Sene A. A., Saharkhiz N., Sohrabi M. R., Moghimian F. (2012). N-acetylcysteine as an adjuvant to clomiphene citrate for successful induction of ovulation in infertile patients with polycystic ovary syndrome. *The Journal of Obstetrics and Gynaecology Research*.

[10] Sacchinelli A., Venturella R., Lico D. (2014). The efficacy of inositol and N-acetyl cysteine administration (Ovaric HP) in improving the ovarian function in infertile women with PCOS with or without insulin resistance. *Obstetrics and Gynecology International*.

[11] Seow K. M., Lee W. L., Wang P. H. (2016). A challenge in the management of women with polycystic ovary syndrome. *Taiwanese Journal of Obstetrics & Gynecology*.

[12] Naderpoor N., Shorakae S., Joham A., Boyle J., De Courten B., Teede H. J. (2015). Obesity and polycystic ovary syndrome. *Minerva endocrinologica*.

[13] Yang B., Sun Z. J., Chen B. (2016). Statin ameliorates endothelial dysfunction and insulin resistance in Tibet women with polycystic ovary syndrome. *European Review for Medical and Pharmacological Sciences*.

[14] Unfer V., Carlomagno G., Dante G., Facchinetti F. (2012). Effects of myo-inositol in women with PCOS: a systematic review of randomized controlled trials. *Gynecological Endocrinology*.

[15] Artini P. G., Di Berardino O. M., Papini F. (2013). Endocrine and clinical effects of myo-inositol administration in polycystic ovary syndrome. A randomized study. *Gynecological Endocrinology*.

[16] Costantino D., Minozzi G., Minozzi F., Guaraldi C. (2009). Metabolic and hormonal effects of myo-inositol in women with polycystic ovary syndrome: a double-blind trial. *European Review for Medical and Pharmacological Sciences*.

[17] Kim M.-J., Lim N.-K., Choi Y.-M. (2014). Prevalence of metabolic syndrome is higher among non-obese PCOS women with hyperandrogenism and menstrual irregularity in Korea. *PLoS ONE*.

[18] El Hayek S., Bitar L., Hamdar L. H., Mirza F. G., Daoud G. (2016). Poly cystic ovarian syndrome: an updated overview. *Frontiers in Physiology*.

[19] Pasquali R., Gambineri A., Pagotto U. (2006). The impact of obesity on reproduction in women with polycystic ovary syndrome. *BJOG: An International Journal of Obstetrics and Gynaecology*.

[20] Carlomagno G., Unfer V. (2011). Inositol safety: clinical evidences. *European Review for Medical and Pharmacological Sciences*.

[21] Minozzi M., D'Andrea G., Unfer V. (2008). Treatment of hirsutism with myo-inositol: a prospective clinical study. *Reproductive BioMedicine Online*.

[22] Papaleo E., Unfer V., Baillargeon J.-P. (2007). Myo-inositol in patients with polycystic ovary syndrome: a novel method for ovulation induction. *Gynecological Endocrinology*.

[23] Papaleo E., Unfer V., Baillargeon J.-P., Fusi F., Occhi F., De Santis L. (2009). Myo-inositol may improve oocyte quality in intracytoplasmic sperm injection cycles. A prospective, controlled, randomized trial. *Fertility and Sterility*.

[24] Gerli S., Papaleo E., Ferrari A., di Renzo G. C. (2007). Randomized, double blind placebo-controlled trial: effects of Myo-inositol on ovarian function and metabolic factors in women with PCOS. *European Review for Medical and Pharmacological Sciences*.

[25] Facchinetti F., Bizzarri M., Benvenga S. (2015). Results from the International Consensus Conference on Myo-inositol and d-chiro-inositol in Obstetrics and Gynecology: the link between metabolic syndrome and PCOS. *European Journal of Obstetrics & Gynecology Reproductive Biology*.

[26] Corti R., Flammer A. J., Hollenberg N. K., Luscher T. F. (2009). Cocoa and cardiovascular health. *Circulation*.

[27] Grassi D., Necozione S., Lippi C. (2005). Cocoa reduces blood pressure and insulin resistance and improves endothelium-dependent vasodilation in hypertensives. *Hypertension*.

[28] D'Anna R., Santamaria A., Cannata M. L. (2014). Effects of a new flavonoid and Myo-inositol supplement on some biomarkers of cardiovascular risk in postmenopausal women: a randomized trial. *International Journal of Endocrinology*.

[29] Santamaria A., Giordano D., Corrado F. (2012). One-year effects of myo-inositol supplementation in postmenopausal women with metabolic syndrome. *Climacteric*.

[30] Zahiri Z., Sharami S. H., Milani F. (2015). Metabolic syndrome in patients with polycystic ovary syndrome in Iran. *International Journal of Fertility and Sterility*.

